# Review of Biochar Properties and Remediation of Metal Pollution of Water and Soil

**DOI:** 10.5696/2156-9614-10.27.200902

**Published:** 2020-08-19

**Authors:** Abudu Ballu Duwiejuah, Abdul Halim Abubakari, Albert Kojo Quainoo, Yakubu Amadu

**Affiliations:** 1 Department of Ecotourism and Environmental Management, Faculty of Natural Resources and Environment, University for Development Studies, Tamale, Ghana; 2 Department of Biotechnology, Faculty of Agriculture, University for Development Studies, Tamale, Ghana; 3 Department of Horticulture, Faculty of Agriculture, University for Development Studies, Tamale, Ghana

**Keywords:** biochar properties, toxic metal contaminants, pyrolysis, remediation, soil and water environment

## Abstract

**Background.:**

Mining, waste disposal, and agrochemical residues have contributed to pollution of water and soil with toxic metals in most low- and middle-income countries, raising concerns of ecological safety and public health. This has prompted many studies into the production and utilization of biochars to adsorb toxic metal contaminants from soil and water.

**Objective.:**

The present study presents a review of biochar properties, the mechanisms of toxic metal adsorption onto biochar, and sorption of toxic metal contaminants in water and soil in small scale applications and laboratory experiments.

**Methods.:**

A total of 305 articles were collected, and after screening for relevance, a final of 164 articles from both high- and low- and middle-income countries were used in this review paper.

**Discussion.:**

Biochar for sorption has proven effective and its raw materials are readily available, cost effective, environmentally stable and a good form of waste management.

**Conclusions.:**

Different techniques of biochar production influence the properties of biochar and adsorption of toxic metals from water and soil.

**Competing Interests.:**

The authors declare no competing financial interests.

## Introduction

Biochar has a carbon matrix structured with a high degree of porosity and extensive surface area, permitting it to act as a sorbent and play a key role in controlling pollutants in the environment.^1^,^2^ Biochar can help farmers increase farm land productivity, concurrently lessening the environmental footprint of farming practices by substituting for chemical pesticides, herbicides and fertilisers.^3^

Biochars derived from animal wastes such as diary manure and broiler litter can immobilize cadmium (Cd), copper (Cu), lead (Pb) and nickel (Ni).^4^,^5^ The immobilization of Cd, Cu, Pb, and Ni in water and soil by biochar derived from broiler litter has been attributed to pi electrons (C=C) and cation exchange.^5^ High Pb affinities by biochar derived from dairy manure are mostly attributable to precipitation of carbonate minerals and Pb phosphate.^4^ The high sorption ability of biochar can be attributed to three processes: electrostatic interactions between a carbon surface that is negatively charged and metal cations; ionic exchange among metal cations and ionizable protons at the acidic carbon surface; and sorptive interaction concerning delocalized carbon electrons.^6^ In the sorption process, oxidation-reduction is possible. For example, removal of chromium (Cr) (VI) by biochar derived from sugar beet tailings is a result of Cr (VI) to Cr (III) ion reduction and its subsequent complexation with the biochar.^7^

Most studies have been conducted on the single metal sorption onto biochar, but potentially toxic metals such as Cu, Cd, Pb, and zinc (Zn) often coexist in polluted water.^8^ Their interactions and associations with other environmental components and with one another are known to influence their mobility.^9^ Hence, it is necessary to understand the mechanisms involved and develop an effective adsorbent that can be immobilized or remove toxic metals from multi-metal polluted water and soils.

Furthermore, biomass thermal properties or process conditions such as atmosphere, heating rate, pre and post treatment, residence time, reactor temperature, reactor type, and pressure, among others, also influence biochar properties.^10^ Based on these factors, biochar properties can differ to a larger extent in terms of their adsorption capacity surface area, ash content, cation exchange capacity, elemental composition, nutrient content, pore size, stability, toxicity, and surface properties (chemical and physical) among others.^10^ Pore-size distribution, porosity, and biochar total surface area have superfluous importance for a range of special effects on adsorption capacity, soil properties and soil microorganisms.^10^ According to the European Biochar Certificate, physico-chemical properties such as biochar ash content, moisture content, yield, pH value, bulk density and total organic carbon content have the greatest effect on biochar metal ion retention.^11^

The functional groups present on the biochar surface impart adsorption potential for toxic constituents such as manganese (Mn) and aluminum (Al) in acidic soils, and Ni, Cu, Cd, lead (Pb), and arsenic (As) in polluted soils.^12^,^13^ Hence, the possible removal or reduction of toxic metals from pollution sources can be achieved using biochar. However, properties of biochar are highly variable, and quality is also affected by feed stock materials, reactor types and pyrolysis conditions. It is necessary to understand the mechanisms involved to develop an effective adsorbent that can immobilize or remove toxic metals from multi-metal polluted water and soil. This review presents an update on biochar properties and remediation of toxic metal polluted water and soil in both small-scale and laboratory experiments.

## Methods

A search strategy protocol was established by the authors prior to conducting the review and refined with the help of previous reviews. The Preferred Reporting Items for Systematic Reviews and Meta-Analyses (PRISMA) 2009 checklist was also used as guide in writing this review article.^14^ A thorough search of the literature was conducted to collect suitable and credible information from reliable sources, including international journals that are reputable, useful reports and books. The scientific papers were appraised through a 2-stage process. In the first stage, the title and abstract of the papers were searched to determine relevance based on the objective of this review. For the second stage, the full body of the articles that were deemed potentially important were reviewed. An initial 305 peer reviewed articles were collected, and a final 164 were selected (dated from 1998 to 2019) and synthesized based on the relatedness to the topic. Google search was used to check for the latest related publications on the topic.

**Abbreviations**
PRISMA: Preferred Reporting Items for Systematic Reviews and Meta-Analyses

### Search design and data collection

Systematic searches were performed for research articles, books, reports and conference papers and abstracts in Science Direct, Springer, Elsevier, and Google Scholar databases on the 25^th^ of June 2019 without limitations on publication year. The keywords “biochar”, “characteristics of biochars”, “sorption parameters”, “biochar adsorption mechanism”, “analysis of adsorption mechanism”, “mechanism of interaction amongst toxic metals and biochar”, “metal polluted water remediation using biochar”, “biochar incorporated into soil changes its physical characteristics”, “adsorption kinetics and adsorption isotherms”, “potential risk of biochar application in the environment”, and “stability and aging effect of biochar” were searched to identify relevant articles.

**Figure 1:**
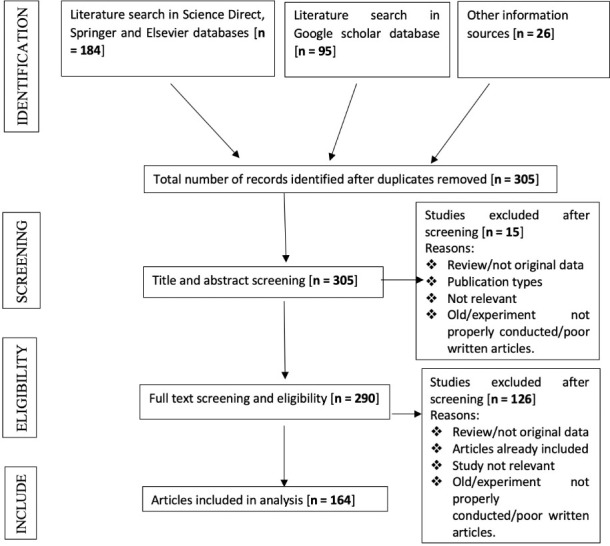
PRISMA flow diagram indicating the number of articles, books and reports that were identified, screened, and included in the current review

The studies included in this systematic review met the following criteria: peer-reviewed research articles, reviews, books, primary reports of research findings on original data, and on the topic of biochar and its properties as outlined above. Only articles written in the English language were considered. Exclusion criteria included: no original data or abstract only; covering only the general properties of biochar, benefits of biochar, role of biochar's potential contribution to climate change mitigation, and bioenergy, full text not included or in languages other than English. The subject matter relevance filtering was done based on title and abstract screening, followed by screening of the full text. Full text screening was carried out in instances where title and abstract were insufficient to determine the relevancy of the article to the present review. Only peer-reviewed research articles, reviews, books and reports were included in the review. The search strategy is illustrated in Figure 1.

### Study quality

Studies sourced from the Science Direct, Springer and Elsevier databases were taken to be of high quality. Most of the articles or reports that were read were excluded, and the most common reasons for exclusion were study already included, review/not original data, publication type, lack of relevance, older study, experiment not properly conducted or article poorly written (*Figure 1*). Overall, we judged journal-published articles to be of higher quality than other information sources. Articles from other information sources often did not adequately describe their experimental design, had inadequate experimental samples, sampling methods, quality control during collection of data, had findings that were largely speculation and lacked firm conclusions, or had poor data analysis.

## Results

A total of 305 articles were collected, and from that total, 164 articles from high-, low- and middle-income countries were synthesized based on the relatedness to the topic *(Table 1).* The information extracted from the studies included author names, year of publication, study design, studied biochar, its properties and remediation of toxic metals, polluted water or soil, and observed response. In the initial database search, 305 articles were considered eligible for extraction of data, as outlined in the PRISMA flow chart *(Figure 1).* A final total of 164 studies were identified through the systematic search and screening. These studies described or are closely related to the subject matter as presented in the PRISMA flow chart *(Figure 1).* This included research articles from Science Direct, Springer, Elsevier, Google Scholar databases and other information sources. The inclusion criteria identified 164 articles, out of which 89.02% were research articles, 5.49% were review articles, 3.05% were books and 1.37% were reports determined to be relevant to the present review after title, abstract and full text screening.

**Table 1:** Included studies by category

**Area of interest**	**Number of studies**
Characteristics of biochars	30
Parameters of sorption	
Effect of pH	10
Effect of initial pH and equilibrium temperature	7
Dosage effect of adsorbent	4
Effect of contact time and initial metal ion concentration on temperature dependent adsorption	2
Biochar adsorption mechanism	16
Organic structure	3
Surface functional groups	3
Surface electrical properties	1
Mineral ingredients	2
Analysis of adsorption mechanism	10
Mechanism of interaction amongst toxic metals and biochar	10
Metal polluted water remediation using biochar	15
Biochar incorporated into soil changes its physical characteristics	24
Adsorption kinetics and adsorption isotherms	11
Potential risk of biochar application in the environment	13
Stability and aging effect of biochar	9

Note: Some studies were included in more than one category

### Data extraction and analysis

We extracted the following data: type of study, author, project year, experimental design, year of publication, methods, type of experiment and publisher for all included studies. Main outcomes were extracted and categorized.

### Biochar characteristics

The pH of biochars could be lowered due to production of phenolic substances and organic acids caused by decomposition in hemicelluloses and cellulose.^15^ Raw almond shell consists of 7.52% extractives, 20.34% lignin, 31.23% hemicellulose, and 38.62% cellulose.^16^

Mineral composition such as calcium (Ca), potassium (K), magnesium (Mg) and phosphorus (P) in biochar and biomass is also responsible for adsorption of metal from aqueous solutions.^17^ Mineral constituents contained in biochar derived from dairy manure are reported to play an essential role in biochar sorption of multi-metals.^18^

Volatile matter and biochar yield are reduced with an upsurge in pyrolysis temperature and heating time owing to the destruction of hemicellulose and cellulose moieties.^19^ The amount of volatile compounds escaping from inside the particle progressively increases during the process of pyrolysis once the final temperature increases.^16^ High ash content of biochar makes it suitable for metal ions and phosphate adsorption.^20^,^21^ In addition, more ash will weaken the biochar's capacity of adsorption toward organic contaminants by masking the active sites of adsorption.^22^

Scanning electron microscopy is a microscopic technique for defining the image macroporosity and physical morphology of solid matter.^23^ Microporous structure may play an important role in contaminant adsorption and water-holding capacity in solution and soil systems.^23^,^24^

Biochars generally have strong aromaticity, larger surface areas, and higher carbon contents, which favors the removal of organic contaminants.^25^ High porosity, large specific surface area, and rich oxygen functional groups on biochar deliver large quantities of active binding sites for organic pollutants via n-π conjugate action, hydrogen bond interaction, ion exchange process, electrostatic adsorption, or other interactions.^25^

Some biochar physical and chemical properties including surface area, pH of materials, porosity, mineral contents, surface charge, and functional groups, play a vital role in explaining the sorption process of metals.^26^ Surface potential, surface area, and pH are significant factors regulating their applications in the environment.^27^

Biochar has many oxygen-containing groups on its surface, and ions could effortlessly outcompete molecules of water for these functional groups for the formation of robust surface complexes.^28^ Metal ion rapid sorption is attributable to the sorbent highly porous structure which offers ready access for great surface area adsorption for the metal ions to the active binding sites.^29^

The adsorption efficiency of biochar tends to be influenced by properties of biochar, like competitive anions, adsorbent dosage, deashing treatment, temperature and pH.^30^ At low pH, biochar functional groups present are positively charged.^30^,^31^

Biochar has high adsorption capacity for metallic pollutants owing to surface heterogeneity.^32^ In addition, many biochars have a high surface area with a network of well distributed pores, including macropores (> 50 nm), mesopores (250 nm), and micropores (< 2 nm).^33^ Biochars with high pore volumes and high surface area have great metal ion affinity as ions can be sorbed physically onto the char surface and retained inside the pores.^34^

Wastewater's multiple components cause interactive effects largely dependent on numerous factors such as metal concentration, pH, quantity of co-cations competing for active binding sites, equilibrium concentration of ions, and quantity and nature of biomass adsorbent.^35^ The preference of an adsorbent for metal ions in multi-metal systems is affected by factors that are linked to the solution's physico-chemical properties such as adsorbent surface properties, temperature, pH and toxic metals properties such as ionic radius, electronic configuration and electronegativity.^36^

Biochar physico-chemical properties (cation exchange capacity, element contents, surface areas, pH and porous structure) are dependent on the temperature of pyrolysis.^37^ During polymerization and the dewatering process, the cellulose and lignin in agricultural residue biochar decay into smaller molecules, and oxygen carbon ratio and hydrogen carbon ratio decrease.^38^ Biochar aromatic structure is used as a π-electron acceptor or donor which form -π bond with contaminants, further impacting biochar adsorption effects on pollutants.^39^ When the temperature of pyrolysis rises, the ester and fat alkyl group are cracked, exposing the aromatic lignin nuclear to the surface, creating a larger surface area.^40^ Biochar produced under high temperature pyrolysis will have strong aromaticity and its toxic metal adsorption is mainly related with the π-bond and specific surface area.^38^ However, more oxygenic functional groups are found in biochar produced under low pyrolysis temperature and its toxic metals adsorption is primarily accredited to complexation with the functional groups.^38^

The temperature of biochar production is a key determining factor of its surface chemistry and properties.^41^ The quantity of either carboxyl groups or mineral in biochar can differ dependent on the original biomass composition and as a function of the pyrolysis conditions employed.^25^ Biochar production is a complicated process of a physico-chemical nature which is affected by the natural inorganic substances and the mechanisms of pyrolysis and interactions of the foremost components like lignin, hemicellulose and cellulose in biomass.^42^ Large quantities of oxygen and carbon in biomass lead to higher biochar yield and functional groups formation such as C-OH, -C-O-R and –COOH.^21^,^22^,^43^

### Sorption parameters

The pH of adsorption media is linked to the sorption of metal—mechanism of the surfaces of the water, revealing the physico-chemical interaction nature of the metal ions in solution and sorption nature of the binding sites.^44^ The pH influences the equilibrium uptake of metal ions in aqueous solution due to the counter reaction of hydrogen proton competition in addition to the sorbent chemistry of the active sites.^45^ One of the main variables affecting sorption process is pH, as it not only influences speciation of the toxic metal ions, but also the ionization degree of the adsorbate and surface charge of the sorbent during the reaction.^30^,^46^,^47^ Reduction in efficiency of adsorption at pH greater than 6 is a result of the soluble hydroxylated complexes formation of the ions and their competition with the binding sites.^48^

Higher biochar pH may be related to higher concentrations of calcium carbonate, alkali metals and alkaline salts.^49^ Biochar produced from high pyrolysis temperatures will be alkaline in nature and can promote toxic metals adsorption and formation of metal hydroxide precipitation and also improve acidic soil.^50^ Naturally, some mine areas have near neutral pH, which is anticipated to result in low mobility of metals.^51^ Biochar has high removal abilities for toxic metals in water/soil owing to its exceptional surface chemistry such as high aromaticity, high surface area, high alkalinity and different functional groups.^52^

### Effect of initial pH and equilibrium temperature

Reaction temperature is a significant factor as it influences process and reaction rate.^53^ Generally, the reaction situations such as cation net release concentration, Cd^2+^ removal capacity and solution pH after equilibrium are similar at different temperatures, without notable differences.^53^ When equilibrium temperature does not considerably affect the adsorption process, it suggests that the adsorption is of a chemical nature rather than physical.^54^ Solution pH is considered the most significant factor of metal adsorption.^55^ Adsorption performance can be affected by initial pH in the following ways: affinity and electrostatic repulsion between adsorbate and adsorbent; the process of ion exchange between adsorbate and adsorbent; and metal ion distribution such as insoluble or soluble and anion or cation.^54^–^58^

### Dosage effect of adsorbent

The increase in adsorbent specific surface area leads to increases of adsorption sites.^59^ In an adsorbentadsorbate equilibrium system, the adsorbent dose is an important factor influencing removal effectiveness.^2^,^60^ Reduction in the amount of adsorption may be attributed to aggregation or overlapping of adsorption sites resulting in declines in adsorbent total surface area.^15^ Increases in the amount of biochar decreased adsorption efficiencies.^2^

### Effect of contact time and initial metal ion concentration on temperature dependent adsorption

Adsorption rate is a function of the initial concentration of a toxic metal, which makes it a significant factor to be examined for sorption effectiveness.^61^ The temperature of the medium is also an effective factor in adsorption efficiency.^62^

### Biochar adsorption mechanism

Biochar physico-chemical properties vary with the type of raw material, means of pyrolysis, feed stock particle size, time of pyrolysis, modification conditions, and temperature.^53^,^63^,^64^ Although biochar structure is affected by several factors, generally, biochar has copious functional groups on its surface, a developed pore structure, high specific surface area and stable molecular structure, with good performance of adsorption, which favors adsorption of contaminants in wastewater.^1^,^65^ The interactions between biochar and toxic metals involve precipitation and adsorption.^50^,^66^,^67^ Weak binding between toxic metals and biochar results in easy separation and desorption.^68^

In biochar production and utilization, many variables including feedstock types and pyrolysis conditions may affect its environmental management efficacy.^69^ Biochar properties differ widely because of the different pyrolysis conditions and raw materials, and it is important to optimize systems of production to yield specifically designed biochar for remediation work.^70^ The specific type of pollutant also affects the ability of biochar adsorption.^71^ The feed stock types and pyrolysis conditions significantly change the physico-chemical characteristics such as polarity, atomic ratio, surface area, element composition, pH, and thus the overall surface characteristic of the biochar.^50^,^72^–^74^

### Organic structure

Biochar organic structure is composed of two layers such as aromatic structures and stacked graphene layers which are interspersed with a graphene layer, armoring the biochar with the rich pore structures and large specific surface area characteristics.^75^ Rich pore structures support the adsorption of organic matter with identical molecular weight, and great specific surface areas improve the physical capacity of biochar for adsorption.^53^ For example, bamboo biochar has a nearly 90% mesoporous structure which aids adsorption of quinolone antibiotics.^76^

### Surface functional groups

Zhang *et al.* showed that when pyrolysis temperature is increased to 500°C or higher, CH (carbon hydrogen) and OH (hydroxyl) on the biochar surface derived from sludge is destroyed.^77^ The surface of biochar derived from chicken manure improved by ammonia/nitric acid can form fresh functional amino groups, which could improve dimethyl sulfide sorption performance.^78^ Biochars derived at 400°C are highly polar in aromatic π-systems and are rich in electron-drawing functional groups.^79^

### Surface electrical properties

Magnesium salt was used to modify corncob biochar to have positive surface electricity, which enhanced adsorption phosphate efficiency.^80^

### Mineral ingredients

Inyang *et al.* found that Pb (II) reacted with dihydrogen phosphate, bicarbonate, and carbonate ions on the biochar surface, enabling Pb carbonate hydroxide, Pb (II) carbonate, and pyromorphite (Pb_5_(PO_4_)_3_X{S}, where X can be bromide, chloride, fluoride, or hydroxide) precipitation.^81^ Generally, organic contaminant adsorption by biochar occurs mostly through the mixture of functional group electrostatic attraction and pore immobilization.^82^

### Analysis of adsorption mechanism

A study found that with greater efficiency of removal of biochar, after equilibrium, the pH and concentration of released Ca^2+^ are higher, which is linked to exchangeable cations of alkaline earth metals that are divalent and soluble alkaline substances.^53^ Gurgel and Gil stated that pH value influences adsorbent surface charge, adsorbate form and ionization state.^83^ Adsorption increased with increasing pH solution as more metal binding sites could be uncovered with negative charges, henceforth ensuing ion attraction with positive charges and adsorption occurring on the surface of the cell.^84^ On the surface of biochar derived from peanut husk, there exist diverse active functional groups (OH and COOH).^50^,^85^ At low pH, protons are present and high in concentration in the system of reaction which protonates on the surface of the adsorbent functional groups and result in electrostatic repulsion amongst the positively charged ion and protonated functional groups.^86^,^87^ Another adsorption mechanism of metal ions might depend on the physical characteristics of biochar such as surface area and porosity.^87^,^88^ Pyrolysis temperature impacts sorption affinities to toxic metals and structural characteristics of biochars.^33^,^89^

### Mechanism of interaction amongst toxic metals and biochar

Key biochar properties such as carbon, ash contents, surface area and pH can be affected by way of post-treatments and thus heighten the ability of biochar to immobilize toxic metals.^90^ Adsorption of toxic metals on biochar surface has been established on multiple occasions using scanning electron microscopy.^91^,^92^ The sorption was attributed to toxic metals complexation with diverse functional groups existing in the biochar, on account of the toxic metals exchange with cations connected with biochar such as Mg^+2^ and Ca^+2^, sulfur, K^+^ and sodium ion, or as a result of adsorption that is physical in nature.^92^,^93^ Moreover, oxygen functional groups are noted to stabilize metal ions in the surface of biochar mostly for softer acids like Cu^2+^ and Pb^2+^.^93^

Furthermore, Mendez *et al.* noted that sorption of Cu^+2^ was associated with the higher oxygenated functional groups in addition to elevated superficial density charge, high average pore diameter and Mg^+2^ and Ca^+2^ biochar exchange content.^94^ Other compounds existing in the ash, for instance carbonates, sulphates or phosphates can also help to stabilize toxic metals by means of precipitation.^4^,^95^ The pH value of biochar increases with pyrolysis temperature, which has been linked with a higher percentage of ash.^96^,^97^ The mobility of toxic metals can also be lessened by biochar by means of changing their redox state.^98^ For instance, the addition of biochar can cause the transformation of Cr^+6^ to Cr^+3^, making it less mobile.^98^ Although the contribution of diverse mechanisms to toxic metal immobilization by different biochars remain unknown, it typically has an effect on pH.^99^

### Metal polluted water remediation using biochar

A study compared eight biochars produced from alfalfa stems, broiler litter, corn stover, corn cobs, guayule shrubs, guayule bagasse, soybean straw, and switch grass activated for their capability to adsorb Zn^2+^, Ni^2+^, Cu^2+^ and Cd^2+^, from water.^100^ Copper ions have a stronger affinity for biochar compared to other divalent metals, due to the surface that forms complexes amongst Cu^2+^ and OH and COOH functional groups onto the biochar.^101^ A study on the efficiencies of removal of Pb ion reported 85% removal by chaff, 86% removal by sun flower husks, 98% removal by tea waste, 90% removal by rice husks and 100% removal by sesame husks.^102^

Dairy manure biochar and rice husk biochar were used for concurrent removal of Cd, Cu, Pb, and Zn from aqueous solution. The study showed biochar produced from dairy manure was more effective in the removal of the four metals than rice husk biochar.^103^ This was attributable to all metals competing for only the ionized phenolic-O groups in the rice husk biochar whereas metal removal by dairy manure biochar resulted from both complexation with ionized hydroxyl-O group and phosphate and/precipitation with metals, resulting in lower competition.^103^

The adsorption of multi-metals onto biochar can be attractive and economical for remediation of metal-contaminated natural acidic solutions like acid mine water.^104^ A single-pollutant systems study reported that biochars (bamboo, hickory wood, sugarcane bagasse and peanut hull-derived) adsorbed 4% to 16% of Cu (II), 11% to 18% efficiency for Cd (II) and 18% to 35% for Pb (II). It concluded that Cd and Pb adsorption was influenced by biochar pore structure, while removal of Cu was influenced by the biochar surface functional groups.^105^ Almost 100% removal of Cd^2+^ was achieved using biochar produced from municipal sewage sludge at 500°C to 900°C, especially the biochar produced at 800°C and 900°C in the initial concentration that ranged from 0 to 50 mg/l of Cd^2+^.^106^ The capacity and mechanism of Cd of adsorption on orange peel biochar produced at various pyrolysis temperatures and 2 and 6 hours heating times were studied. The study showed about 80.6% to 96.9% removal of Cd^2+^ occurred within the first minute of contact in the solution.^107^

Cadmium removal efficiency by biochars (three) were greater than 90% at an optimum pH value of 5, with the highest stretch to 99.24%.^108^ Sunflower seed husk biochar showed a strong ability to remove most Cu^2+^, while about 74.15 to 81.00% of Cu^2+^ was removed by sunflower seed husk feedstock within 5 minutes of the batch experiment in the tested temperature range. However, a gradual increase of Cu^2+^ removal of 85.74% to 89.40% by sunflower seed husk feedstock was observed after 96 hours of reaction.^109^ The removal efficiencies of Cd using different materials from aqueous solution showed that among the different materials, mushroom waste recorded the lowest efficiency of 38.7% for Cd, 86.6% for soybean straw and peanut husk biochar showed significantly higher removal efficiency of 99.2%.^110^ Percentage removal of Pb (II) was up to 99% in 40 minutes from aqueous solution using biochar produced from bamboo and calcium sulphate.^111^ A study found that biochar was able to remove 96.88% Cd^2+^, 96.23% Zn^2+^, 95.96% Co^2+^, 93.38% Cu^2+^ and 88.79% Pb^2+^ ions in acidic solutions.^112^ Removal efficiency of Cd, mercury (Hg) and Pb by groundnut and shea nut shell biochars in a mono-component system was almost 100%, over 99.60% for Cd and 100% for Hg in a binary system, and Cd versus Pb, and Hg versus Pb were almost 100%, and in the ternary system the removal was greater than 97.50% in the aqueous phase.^113^ Higher adsorption capacities of Pb^2+^ and Cd^2+^ of dairy manure biochar have been reported to be 68.08 mg/g by Cd and 175.53 mg/g by Pb.^114^

### Biochar incorporated into soil changes its physical characteristics

Biochar incorporated into soil changes its physical characteristics, for instance density, structure and pore size distribution, with implications for soil aeration, soil workability and water holding capacity.^115^, ^116^ Application of biochar into soil showed an upsurge in the general net surface area of the soil and subsequently, may improve nutrient and retention of soil water and soil aeration predominantly in soils with fine texture.^117^–^119^ The alkaline properties of biochars increased the pH of solution, which encouraged metal immobilization through a decrease in metal solubility and metal precipitation.^120^

The addition of biochar did not result in the total toxic metal content decrease in soil, nevertheless, addition of biochar reduced the mobility of Pb, Cr and Cd and the bioavailability of Zn, Pb and Cd. Park *et al.* reported the effect of two biochars in a toxic metal spiked soil and a strongly contaminated natural soil.^121^ Uchimiya *et al.* reported that biochar derived from manures with a low or high ash proportion or phosphorus were less effective in toxic metal immobilization.^122^

Beesley *et al.* found that after the addition of biochar, As can upsurge in soil pore water, but is reduced during transfer to plants.^123^ Karami *et al.* reported that biochar incorporation into mine soil contaminated with Cu and Pb showed that addition of biochar reduced pore water levels of Pb to half of their concentrations in the mine soil.^124^ Jiang *et al.* established that fractions of acid soluble Cu^2+^ and Pb^2+^ diminished by 19.7% to 100%, and 18.8% to 77%, respectively, dependent on the amount of biochar.^125^ Performing acid or additional oxidant post-treatment increases oxygen-containing surface functional groups (hydroxyl, carbonyl and carboxyl) and this plays an important role in sorption of metal ions on biochar.^126^

Treatments comprised of earthworms and biochar did not result in plant availability or higher mobility of metal.^127^ As a significance of metal immobilization, biochars can lessen polluted soils phytotoxicity, resulting in increases in root length and germinated seeds percentage.^128^ Cui *et al.* established that Cd uptake was reduced in cropped wheat and paddy fields in soil.^129^ Zheng *et al.* reported Zn, Pb and Cd to have been reduced on rice shoots, especially when using biochar derived from straw.^130^ Biochar can be used to recover polluted areas and brownfields as it improves growth of trees, crops and other flora and increases soil fertility.^3^ Biochar can also reduce the quantity of toxic metals and other contaminants in soil and averts them from delivery into water bodies.^131^

Biochar presence in the mixture of soil has a significant effect on the system's physical nature, depth, porosity, texture, consistency, and structure during the course of changing pore size distribution, packing, particle size distribution, surface area and bulk density.^132^ The penetration depth and availability of air and water into the root zone is mostly determined by the physical compositions of soil horizons.^133^

The biochar surface can contain numerous chemically active groups, such as ketones, COOH, and OH that engender a great potential for toxic chemical adsorption in metal-contaminated soils.^12^,^13^ Particles of biochar promote air flow and increase soil porosity through landfill cover and, as a consequence, diffusion of dioxygen increased within the landfill cover, resulting in higher microbial degradation levels.^134^ Biochar can be used as a soil conditioner by enhancing the biological and physical characteristics of soils, for instance water holding capacity and retention of soil nutrients, while also supporting plant growth.^135^ Furthermore, the leaching of soil nutrients are reduced by biochar, while promoting nutrient availability for plants and decreasing the bioavailability of toxic metals.^136^ Hossain *et al.* stated that biochar derived at low temperatures of 300°C or 400°C is acidic in nature and alkaline at high temperatures of 700°C.^137^

### Adsorption kinetics and adsorption isotherms

Kinetic studies provide understandings of functional groups of biochar interactions with contaminants.^138^ Furthermore, kinetic studies have significant importance in determining the required resident time for process-scale up and complete adsorption reaction.^139^ To improve the interaction by means of greater ionic concentration, other treatments, for example sodium sulfite, iron(III) oxide, and iron(II) sulfate on the surface of biochar, were examined to improve precipitation and chemisorption for removal of toxic metals.^140^ Kinetics adsorption showed the best fit in a pseudo second-order model that indicates that the process of adsorption was largely governed by available active sites on the surface of biochar instead of contaminant concentrations.^30^,^141^ The best kinetic model for most of the inorganic and organic pollutants was a pseudo second-order model.^142^ The studies indicated that physisorption is rapid and generally happens on carbon inner pores, while chemisorption is a limiting rate step which occurs slowly on the surface of carbon.^143^ Conversely, studies have revealed that biochar maximum removal efficiency was a result of the contribution of both mechanisms of physisorption and chemisorption.^30^

The Langmuir model assumes an adsorption monolayer with no interactions among the molecules adsorbed, whereas the Freundlich model illustrates the process of chemisorption on the biochar heterogeneous surface.^144^ Distribution of surface charge on a biochar surface is largely dependent on pH, and conversely doses affect the capacity of adsorption and ion concentrations affect the abilities of systems for metal removal.^144^ Langmuir isotherm was also used to establish the removal of toxic metals using biochar by chemical treatments via increasing ionic concentrations, which indicated greater efficiency than untreated biochar.^144^

### Potential risk of biochar application in the environment

Depending on the feed stocks used in their production, some biochars may contain some toxic metal pollutants (for example Cd, Cu and Pb) or other toxic metals, which chiefly originate from toxic metals-containing feed stocks such as industrial solid waste, sewage sludge, and residue for production of biogas.^145^ Biochar can induce both negative and positive effects on microorganisms, fauna and plants.^146^ For agrochemicals like herbicides and pesticides, biochar has a strong capacity for their adsorption, and also causes deactivation or accumulation of herbicides and pesticides, and residues act as a secondary pollutants.^69^

The inherent physico-chemical properties and structure of biochar have indirect or direct impacts on the soil micro environment by affecting water content, nutrient content, porosity, soil bulk density and cation exchange capacity.^147^ Moreover, biochar can alter soil aeration and moisture condition, and affect the potential of soil redox, changing some charge-sensitive toxic metals toxicity.^148^

The effects of biochar on soil physico-chemical properties vary by type of feed stock, soil type, aging and biochar application rate.^149^ Biochar can be polluted by Ni and Cr during the process of pyrolysis owing to reactor stainless-steel erosion parts.^150^ The effects of biochar on the treatment of toxic metal-contaminated soils also change with the species of metal and different physico-chemical properties of soils such as clay content, cation exchange capacity, pH value, redox potential, nutrient balance, concentrations of trace elements, moisture, anaerobic or aerobic conditions, temperature and soil organic matter content.^151^ Conversely, the two most important factors affecting toxic metals bioavailability are soil organic matter content and pH.^152^ Biochar that is alkaline can induce liming effects in soil and cause metal immobilization.^50^ Biochar addition into soils can lead to pH increase and metal solubility decline.^153^

Cadmium and Pb adsorption can be affected by biochar pore structure, while removal of Cu may be connected to surface functional groups, which could enhance the ability to bind metals and promote complex formations.^154^ Biochar ash content can also affect biochar sorption behavior, promote metal immobilization and precipitation.^152^

### Stability and aging effects of biochar

Biochar has a high degree of aromatization structure and carboxylate esterification, very low solubility, high carbon content, high stability, high boiling point, and strong resistance to chemical, physical and biological decomposition.^155^ These characteristics allow it to exist for thousands of years in soil under natural environmental conditions.^156^ Adsorption ability, surface functional groups, and other biochar properties change with respect to time by microbial degradation or oxidation, which have been identified as processes of biochar aging.^157^ Furthermore, the long-time stability of biochar must be further demonstrated in field studies.^158^ The long-term effects of biochar application on soil health and function, including its effects in different soil types, remain to be fully eluciated.^159^

Some studies on immobilization capacity and properties of biochar showed changes in biochar properties under simulated processes of short-term aging such as physical aging, acid aging, and hydrogen peroxide aging.^160^ Biochar alkalinity can reduce aging, change the mineral composition, and increase the number of oxygen-comprising functional groups (for example phenols and ketones).^38^,^161^ Six-month aging in soil increased the number of grain husk biochar oxygenated functional groups and hence facilitated Zn binding onto biochar.^162^

## Discussion

Feed stock composition is responsible for the differences in pH across various biochars. The pH of biochar could be lowered due to production of phenolic substances and organic acids caused by decomposition in hemicelluloses and cellulose.^15^ The pH of biochars are mostly alkaline in nature and tend to upsurge with increasing pyrolysis temperature.

The nature of feed stock and pyrolysis conditions of biochar influence its mineral composition. Biochars with higher mineral compositions provide extra opportunities for adsorption of toxic metals from water. Mineral composition such as Ca, K, Mg and P in biochar and biomass is also responsible for adsorption of metals from aqueous solutions.^17^ However, the role of mineral components in biochar is not well articulated, and a greater understanding is needed of the bioavailability of toxic metal ions in some biochars, the role of biochar intrinsic minerals, and the design of specific applications by integrating appropriate minerals into biochar.

The wide diversity of feed stocks is one of the main factors affecting biochar properties as it endows biochars with diverse chemical structures and compositions. For instance, the formation of porous biochars is significantly influenced by agricultural waste structures.^25^ The average pore size of biochar decreased with temperature of pyrolysis, as large pores are destroyed during further heating, causing the formation of more small pores.

Pyrolysis temperature influences biochar's aromaticity, morphological, structural and elemental properties. Many studies showed that different pyrolytic temperatures and feed stocks affect the pH, carbon content, aromaticity and ash content of biochar, among other parameters, which can further impact the effectiveness of biochars in repairing metal pollutants.^5^ Many biochars can sorb positively charged metal ions through electrostatic attractions because they are negatively charged surfaces. Biochar produced at various temperatures are expected to have diverse properties which will influence their individual performance in toxic metals immobilization/removal in contaminated soil and water. The selection of feed stock and pyrolytic conditions of biochar is critical to the removal of toxic metals from water and soil. Feed stock characteristics and pyrolysis conditions largely control the physico-chemical characteristics of biochar which regulate a given application suitability in addition to defining biochar behavior, fate and transport of contaminants in the environment.

The pH influence on adsorption is largely dependent on biochar type and the target pollutants. pH influences the equilibrium uptake of metal ion in aqueous solution due to the counter reaction of hydrogen protons competition in addition to the sorbent chemistry of active sites.44 Soil pH is exposed to temporal variations which affect the adsorption of toxic metals. In addition, the usage of ammonium fertilizers, nitrogen fixing species and acid rain can lower soil pH, occasioning an increase in metals mobility and related environmental risks. The alkaline minerals present in biochar when applied to soil or water will increase the pH of the environmental media. In addition, alkaline minerals on the biochar surface also become hot spots for toxic metal surface precipitation. Depending on the nature of agricultural waste used for the production of the biochar, these properties vary from biochar to biochar.

The pH after equilibrium can advance to a sufficient level by the biochar buffer capacity of pH at a higher initial pH, and the ion transforms to precipitation of hydroxide to settle. Adsorption performance can be affected by initial pH in the following ways: affinity and electrostatic repulsion between adsorbate and adsorbent; the process of ion exchange between adsorbate and adsorbent; and metal ion distribution such as insoluble or soluble and anion or cation.^54^–^58^

The adsorbent dosage has a significant influence on adsorption efficiency.^2^,^60^ Applying an optimum biochar dosage for pollutant removal is critical to its low-cost application. The adsorption of toxic metals increased with upsurge in the adsorbent dosage. Increases in the amount of biochar decreased adsorption efficiencies.^2^ Reduction in the amount of adsorption may be attributed to aggregation or overlapping of adsorption sites resulting in declines in adsorbent total surface area.^15^ Initial upsurge in the adsorption percentage can be accredited to the availability of more adsorption sites and increased adsorbent surface area. Conversely, not all adsorption sites are available for exchanging or binding on account of aggregation and overlapping, so a portion of the sorbent will not be used, and declines as the dosage upsurges after a certain value.^53^

The energy of the system facilitates ion attachment onto the cell surface at higher temperatures. The adsorption increase with temperature may be accredited to an upsurge in the number of active binding sites available for the adsorption on the sorbent.^46^

Biochar, with void structures and high carbon content, has copious aromaticity oxygen-comprising functional groups. Ionic/nonionic and polar/non-polar organic pollutants have diverse affinities for biochar when compared to anionic and cationic metals.^50^ Adsorption efficiency can be improved for soil and water contaminants if the properties of biochar and water and soil properties are well understood prior to remediation interventions. For soil remediation, moderately strong binding is favored for the low leachability/bioavailability of toxic metals in soil and long-standing stability.^68^ The effect of adsorption of toxic metal ions onto biochar is influenced by various factors such as pyrolysis temperature, biochar feed stock, soil pH, chemical and physical properties of metal ions and the dosage of biochar addition. Biochar applications in the environment are highly dependent on its mechanisms of interaction with toxic metals. Not all biochars are effective for adsorption of pollutants in the environment.

The predominance and variability of a specific reaction of a biochar are controlled by its specific physico-chemical characteristics, which is attributed to pyrolysis conditions and feed stock type. Variation in the characteristics of biochar greatly influence its efficacy and suitability for the remediation of targeted contaminants.

Biochar surface functional groups fix metal ions by complexation, surface precipitation, and electrostatic attraction. For instance, Zhang *et al.* showed that when pyrolysis temperature is increased to 500°C or higher, CH (carbon hydrogen) and OH (hydroxyl) on the biochar surface derived from sludge are destroyed.^77^ Poor functional groups as well as electron-rich groups are present in biochars derived at high temperatures.

Biochar surface electrostatic attraction plays a very significant role in contaminant adsorption. Generally, biochar surface electricity is negative, making adsorption performance good for positive ions such as metal ions and ammonia, among others. For instance, magnesium salt was used to modify corncob biochar to have positive surface electricity, which enhanced adsorption phosphate efficiency.^80^ The modification of biochar to have positively charged surface electricity can be used to adsorb anions.

Mineral components of biochar can increase the properties of adsorption. The adsorption of toxic metals mainly through complexation reaction, ion exchange and electrostatic attraction of surface functional groups, in addition to the adsorption of nitrogen, mineral components precipitation, and phosphorus occurs mostly through the blend of electrostatic attraction with mineral composition precipitation.^82^

Adsorption increased with increasing pH solution as more metal binding sites could be uncovered with negative charges. This phenomenon was attributable to the competition of substantial protons for vacant binding sites of adsorbents for the adsorption at lesser pH values. The organic matter contribution on Cd adsorption is small; alkaline earth metals, for instance Ca^2+^, are essential for adsorptive reaction, and precipitation formed between Cd^2+^ and Ca^2+^ by means of ion exchange and rising pH may be two main adsorption routes.^53^ The toxic metal ion sorption reaction should be adaptable to a varied range of pH and is reliant on biochar characteristics determined by feed stock materials, pyrolysis temperature and conditions.

Characteristics of biochar are a function of many factors, including particle size, type of feed stock, and pyrolysis conditions and temperature. Several studies investigated the impact of pyrolysis temperature on sorption affinities to toxic metals and structural characteristics of biochars.^33^,^89^ The mechanisms of sorption are largely dependent on the presence of cations in soil and biochar. Elevated values of pH after biochar addition can cause precipitation of metal in the soils.

Biochar performed better due to its easy access to functional groups and high surface area. For instance, copper ions have a stronger affinity for biochar, due to the surface that forms complexes amongst Cu^2+^ and OH and COOH functional groups onto the biochar.^101^ The efficiency of metals removal by digested whole sugar beet biochar was above 97%, indicating that biochar had a great affinity for the tested toxic metals.^81^ It can be concluded that all types of biochars were able to remove toxic metals and their removal rate varied due the biomass nature, physico-chemical properties of biochar and the environmental conditions.

The addition of biochar did not result in a total toxic metal content decrease in soil, nevertheless, addition of biochar reduced the mobility of Pb, Cr and Cd and the bioavailability of Zn, Pb and Cd. A sequential extraction of a number of metals and biochar derived from chicken manure was effective at reducing extractable Pb and Cd concentrations, but not Cu concentrations, while biochar derived from green waste was more effective in reducing all of the metal ions.^121^ Biochars produced at 700°C were more effective, which was attributable to the material transformations, including nitrogen removal containing leachable aliphatic and heteroaromatic functional groups.

Biochar has the potential to improve the agronomic value and quality of soil, while minimizing the injurious effects of toxic metals. Some biochars pose no risk of toxic metals increasing in plants and are therefore safe with regard to transfer in the food chain. For instance, treatments comprised of earthworms and biochar did not result in plant availability or higher mobility of metals.^127^ Biochar can be used to recover polluted areas and brownfields as it improves growth of trees, crops and other flora and increases soil fertility.^3^ In addition, it improves the capability of soil to handle flooding and drought. Biochar influences the physical characteristics of soil that may consequently have an uninterrupted effect on plant growth. Furthermore, the leaching of soil nutrients are reduced by biochar, while promoting nutrient availability for plants and decreasing the bioavailability of toxic metals.^136^

Many factors influence the kinetics of adsorption, such as feed stock type, pyrolysis temperature and pH. Biochar amendment improves the pH of environmental media, which has led to higher pollutant removal efficiency. Studies provide information on pollutant interaction with biochar, which facilitate superior understanding of adsorption capacity, type of interaction and functionality.^140^,^146^ Langmuir and Freundlich adsorption isotherms have been employed to establish the relationship between the adsorbate concentration at equilibrium conditions and adsorbate loading on biochar. The Langmuir isotherm is best fitted for inorganic contaminant removal, particularly to study biochar pH, initial ion concentration and dosage effect on removal mechanisms of toxic metals.^146^,^147^,^149^ However, the model best fitted for organic pollutant removal is the Freundlich model, which provides a better understanding of the pyrolysis temperature effect on organic contaminant removal in environmental media.

There are two main potential risks of biochar usage in the environment, the unconscious discharge of toxic elements from materials used in biochar production and from the biochar-based material production process.^25^ When applying biochar in water treatment, these toxic metal pollutants can easily be released into the water, causing secondary pollution. Soil pH is one of the key soil attributes controlling mobility of toxic metals in contaminated sites by influencing other soil properties. Most toxic metals become more bioavailable under acidic conditions. Biochar that is alkaline can induce liming effects in soil and cause metal immobilization.^50^ Biochar application can cause phytotoxicity by toxic metals, as well as low fertility, low water holding capacity, extreme pH values and poor soil structure, preventing establishment of plants.^51^ The effect of biochar on the bioavailability of toxic metals varied with the influence of application rate and raw materials. The two most important factors affecting toxic metals bioavailability are soil organic matter content and pH.^159^ Biochar addition into soils can lead to pH increase and metal solubility decline.^153^

The long-term impacts of biochar on soil microbe/fauna, soil texture, fertility, toxicity and mineralogy are likely to be of greater concern. Understanding the lability and distribution of metal(loid) changes during aging processes is important for implementation of projects involving biochar in practical remediation works. Toxic metals can be released from particles of biochar into soil and interact with soil components, such as minerals.^163^ Some biochars contain toxic metals as a result of the relatively high concentrations of metals in the original feedstock.^164^

## Conclusions

Biochar properties have the ability to effectively adsorb toxic metals and other pollutants in water and soil. However, the characteristics of biochar are largely dependent on the feed stock biomass and pyrolysis conditions. Biochars have been used to adsorb toxic metals, agricultural residues and other organic contaminants. Biochar provides an excellent, eco-friendly and cost-effective medium for controlling water and soil environmental contaminants. However, understanding of the mechanisms of biochar adsorption is crucial for their utilization in water and soil remediation. There is an ever-increasing need for understanding the mechanisms of adsorption, in order to utilize readily available biomass feed stocks.

#### Recommendations

Agricultural waste biochar is an ecofriendly and promising method for the remediation of water and soil contaminated with toxic metals, however a few issues need to be addressed. Biochar has mainly been investigated under laboratory conditions, not in real environments, or scaled up for use in large contaminated sites. Furthermore, no single biochar will be effective in removing or immobilizing all toxic metals in polluted water and soils. The feed stock, pyrolysis temperature and conditions, biochar production, properties, cost and acceptability for use can lead to differences in the remediation effects of toxic metals in polluted water and soil. Hence, for successful application and wide use, there is an urgent need to determine optimum biochar options for toxic metal polluted water and soil with practical applications. Biochar properties vary with time. Further study is needed to understand how biochar influences the water and soil environment.
